# The Influence of Physical Properties and Increasing Woven Fabric Layers on the Noise Absorption Capacity

**DOI:** 10.3390/ma14206220

**Published:** 2021-10-19

**Authors:** Bethalihem Teferi Samuel, Marcin Barburski, Ewa Witczak, Izabela Jasińska

**Affiliations:** 1 Faculty of Material Technologies and Textile Design, Institute of Architecture of Textiles, Lodz University of Technology, 116 Zeromskiego St., 90-924 Lodz, Poland; marcin.barburski@p.lodz.pl; 2 Lukasiewicz Research Network-Textile Research Institute, 5/15 Brzezińska St., 92-103 Lodz, Poland; ewa.witczak@iw.lukasiewicz.gov.pl (E.W.); izabela.jasinska@iw.lukasiewicz.gov.pl (I.J.)

**Keywords:** acoustic barrier, woven fabric, polyester yarn, surface roughness

## Abstract

Noise pollution from the environment may wreak havoc on a person’s wellbeing. Numerous sound-absorbing materials are employed to address these issues, one of which is textile-woven fabrics. In this study, 12 woven textiles with four different weave structures (plain, rib, sateen, and twill) and those formed from three distinct polyester yarns were evaluated for their sound absorption properties using an impedance tube. The study was conducted within the range of 80–5000 (Hz) frequency. Part of the investigation was measuring different layers of woven fabrics under three different measuring conditions. Firstly, only woven fabrics were evaluated. Following that, woven and nonwoven textiles were measured. The third variant, in addition to the woven fabrics, included an air gap. In addition, this study includes tests and analyses of the effect of roughness and porosity of the fabric structure on the effectiveness of noise reduction by woven fabrics. The absorption capacity of plain fabric is higher at lower frequencies than other woven fabrics. Other weave structures noise reduction efficiency is higher as the frequency range increases. The absorption efficiency of plain fabric decreases with fabric layering. Utilizing woven fabric combined with nonwoven fabric reduces noise more effectively than the air gap variant. Low surface roughness and a highly porous surface of the fabric indicate a high noise reduction coefficient (NRC).

## 1. Introduction

The term “absorption of sound” refers to the process by which the energy contained in an incoming sound wave is converted into materials via damping and viscous loss. The sound absorption coefficient (a) of a material is defined as the ratio of the substance’s absorption energy to the incident sound energy. The sound absorption coefficient is the most critical indicator for evaluating the absorption capacity of a material. In addition, the noise reduction coefficient (NRC) must be computed in order to compare the sound absorption effectiveness of various materials. The value of the sound coefficient is directly related to the characteristics of the material, the frequencies of the sound wave, and the direction of incidence [1,2,3].

Sound-absorbing materials play a vital role in securing human wellbeing from the unnecessary noise generated by the environment. The sound absorption characteristics of porous fibrous materials have been extensively researched, and one of them is woven fabric. The porous sound absorption material in general determined by characteristics such as mass per unit area, porosity, pore size, density, and thickness [3,4,5]. The analysis of fabric porosity, area density, pore size, air permeability and thickness on sound absorption were studied [6,7,8]. It was found that the acoustic absorption coefficients were significantly related to air permeability. As a result, lower air permeability in woven and nonwoven fabrics usually means preindication of better sound absorption.

As stated, refs. [4,9,10,11,12] density is a crucial characteristic for porous sound absorption materials. The sound wave may not reach the interior of materials with too closely packed fibers. As a result, the sound wave friction between the pore and the fiber strand is reduced. On the other hand, the energy lost as heat will be negligible if the material’s fiber content is very low. The thickness of the sound absorber material is another important factor that affects effective absorption, especially in the low frequency range. A homogeneous porous material (HPM) with a thickness equal to one-quarter of the sound wavelength was used to demonstrate effective low-frequency noise absorption. Combining isotropic or anisotropic porous screens (1 mm thickness or less) with a slight increase in the thickness of sound-absorbing material can also improve sound absorption [13,14,15].

In porous acoustic textiles, the tortuosity value indicates the flow resistance of the material. The material’s high flow resistivity is due to its long and complex porous structure, which causes it to be high tortuosity. As a result, porosity and the material’s normal thickness is directly proportional to tortuosity [16,17,18].

The cover factor, yarn density, fabric treatment, and the air gap behind the fabric all play a significant role in the sound absorption performance of woven fabrics. However, the intrinsic properties of woven fabrics have a negligible effect on their noise absorption coefficients at low frequencies (f < 500 Hz), they have a noticeable effect at high frequencies (f = 4000 Hz) [3].

In addition to the weave structure of the fabric and the yarn properties, the variation in the number of filaments leads to a variance in sound absorption performance even among textiles with the same weave structure. Additionally, variation in yarn density within the same weave structure produces a different effect. As a result, the number of filament yarn and the density of the yarn are directly related to the effectiveness of a weave type on sound absorption. Furthermore, the paper [9] demonstrates that loosely fibrous patterns improve the sound absorption capabilities of woven fabrics. Inversely, a larger fiber diameter and higher linear density reduce the absorption of sound waves. On the other hand, the angle of sound incidence has been reported to have a significant effect on the effectiveness of sound absorber materials [19,20].

Multilayered materials are often utilized to increase the efficiency of fabric absorption [2,21]. However, when a woven fabric layer is combined with a nonwoven layer, the fabric behaves like a resistive layer, modifying the absorption [22,23]. The study [24] shows that combining woven fabrics with a high noise reflection coefficient together with several layers of nonwoven fabrics demonstrates that the woven fabric provides a significant amount of noise absorption capacity (20–40%) in the low frequency range (f < 500 Hz).

Considering the advantages of woven fabrics’ mechanical capabilities and a variety of aesthetic features, it encourages us to use them as sound absorber materials. Simultaneously, this may expand the range of woven fabric applications. The research presented in this paper is a follow-up study to the previous study’s findings, which are presented in the publication [20]. This paper presents the result of the noise absorption performance of a single layer to several layers of polyester woven fabric using an impedance tube measuring device. The measurement method is applied by combining nonwoven fabric with woven fabric and air gap with woven fabric. A total of 12 polyester woven fabrics were used for this research. Apart from the impedance tube test, this research investigated the effect of the fabric’s surface roughness and porosity on the sound attenuation performance of woven fabrics. The findings and analysis were conducted in order to get a comprehensive knowledge of the sound absorption properties of woven fabric in various configurations. This may aid in understanding the conditions that exist during the manufacturing and application of such material.

## 2. Material

**Figure 1:**
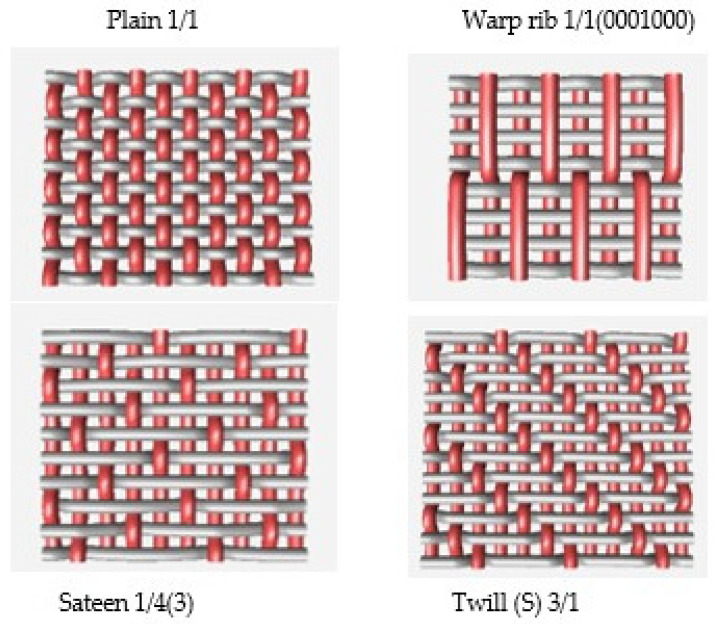
Types of fabric weave interlacement.

Polyester yarn of varying characteristics was utilized in this experiment, such as drawn textured yarn (DTY) dtex 167 × 2 (f 32 × 2), twisted yarn dtex 334 f 32 × 2, (S95), and staple yarn 200 × 2 dtex. The selected four weave structures are presented in Figure 1. In the Lukasiewicz Research Network-Textile Research Institute (Lodz, Poland), 12 fabrics were woven using a Sample Dobby Loom SL 8900 S (CCI Tech Inc., New Taipei City, Taiwan). The measured warp and weft density of yarns together with mass per unit area are presented in Table 1. The fabric mass per unit area was tested according to PN-ISO 3801:1993 [25]. Detailed woven fabrics preparation, technical description, and their analysis were published in the previous paper [20].

## 3. Methods 

### 3.1. Acoustic Test

**Figure 2:**
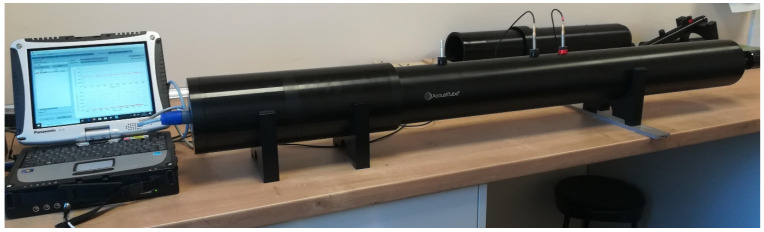
Sound absorption coefficient measurement-impedance tube.

The sound absorption coefficient (*α*) of each sample was determined as a function of the impinging wave’s octaves band frequencies, which was within the range of 80–5000 Hz. According to PN-EN ISO 10534-2:2003, the sound absorption coefficient was measured using the transfer function method [26]. Three measurements were made on each sample in accordance with the specifications of the standard using an impedance tube (Figure 2), and the findings were given as a mean value. The sound absorption coefficient measurements were performed using three different measurement variants. In the first variant only woven fabric was tested, whereas the woven fabric with a nonwoven polyester base (PES) in the second one. Finally woven fabric with a 30 mm air gap between their and the solid plate (Table 2). The nonwoven base thickness was 30 mm, reaching mass per unit area of 623 g/m^2^. In each variant, the arrangement of the woven fabric was into single, double, and triple layers of woven fabrics inside the impedance tube. The woven fabric holder made of acrylic was used as a unique sealing, securing textiles as firmly as possible. In Table 2 and Table 3 the variants for determining the sound absorption coefficient were summarize, showing the number and angle of woven fabric layers inside the tube.

### 3.2. The Percentage Number of Yarn in the Fabric (Volume Porosity)

The percentage number of yarn in the fabric (volume porosity) is a part of the porosity analysis on the fabric surface that indicates how many fibers are in the fabric. The amount of polyester fiber in the fabric volume (Pv- Volume porosity) is calculated as follows;

Pv = Mp/(h × q) × 100%(1)

where:

h—Fabric thickness [m],
Mp—Mass per unit area [kg/m^2^],
Q—Raw material (polymer) density [kg/m^3^] for polyester, 1.38 g/cm^3^ = 1380 kg/m^3^.

### 3.3. Measurement of Surface Roughness Test

**Figure 3:**
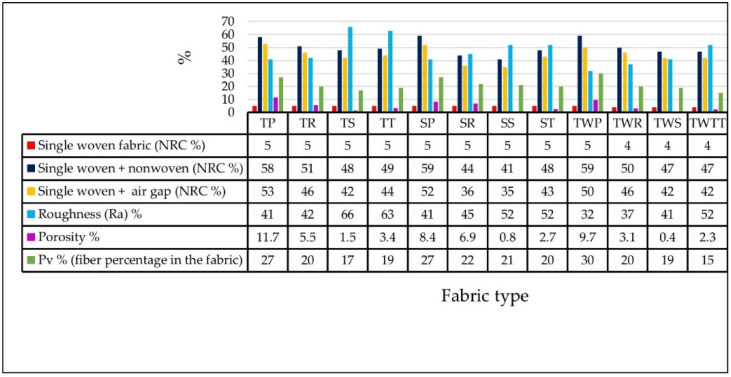
Noise reduction performance of the woven fabric and its relation to the characteristics of the fabric.

Measurements were made using a MicroSpy^®^ profile profilometer with an FRT CWL sensor. A total of 12 woven fabrics were analyzed for roughness index (Ra). The first step was to prepare the square samples 5 cm × 5 cm fitting the MicroSpy^®^ measuring area. Then, the measurements were performed according to the DIN EN ISO 4287 standards [27]. From each sample, three repeated measurements were performed in the warp and weft directions of the fabric. The result in Figure 3 presented the mean value of the sample measurements.

### 3.4. Porosity Test

The optical porosity test was evaluated using Olympus stereoscopic microscope with transmitted light source located inside its table and with a magnification of 10×. The porosity of the sample was evaluated using Test Procedure No. 60, as elaborated in Textile Research Institute. The measurement is based on the quantity of light which pass through the textile sample placed horizontally on the microscope table. The sample was illuminated with passing light at a constant intensity of 1000 lux. The porosity is defined as the proportion of the thresholded area to the total sample image area. The area of sample through which the light passed are usually pores in fabrics structures. The image of samples grabbed in such conditions is thresholded to differentiate pore and solid material areas of woven fabric. The summary area of pores is calculated, giving the percentage of sample areas covered by light-transmitting pores. The test was performed under the normal atmospheric conditions, according to PN-EN-ISO 139:2006/A1:2012, which means a temperature of 20 ± 2 °C and relative humidity of 65 ± 4% [28]. The findings are summarized in Figure 3.

## 4. Results and Discussion

### 4.1. Noise Reduction Properties Comparison

The noise reduction coefficient (NRC) is presented to compare the effectiveness of sound absorption between fabrics. This metric expresses the degree of sound absorption and is proportional to the sound absorption coefficient. The NRC of the fabrics calculated at the mid-range frequencies and the arithmetic average of NRC was at frequencies 250, 500, 1000, and 2000 Hz octaves. The NRC of the samples was calculated at the frequencies at which the human ear was mostly sensitive. A sound-absorption material is generally required to have a noise reduction coefficient (NRC) value greater than or equal to 0.20. Practically useful materials have an NRC higher than 0.4, while materials with an NRC greater than 0.56 are referred to be high-efficiency sound-absorbing materials [3,29,30]. The amount of reflected sound waves from the tested surface are presented in percentages. In Table 4, the noise reduction coefficients (NRC) of the fabrics and reflection of sound incidence are presented according to the different types of measurement variants. The nonwoven fabric has an NRC of 0.21 and a reflectance of 79%. Additionally, the NRC and reflection of the test performed without the sample were 0.028 and 97%.

NRC = (*α*_250_ + *α*_500_ + *α*_1000_ + *α*_2000_)/4 (2)

The NRC results demonstrate that as the layer of woven fabric increases, the noise reduction also increases somewhat in variant I and significantly increasing in variant II and III except for plain fabrics. The result of the II variant demonstrates a substantial increase in NRC to that of the I and II variants. The second variant result is the presence of nonwoven fabric. However, by integrating woven and nonwoven fabrics, the NRC performance of woven fabrics is enhanced; also, the performance of nonwoven sound absorption is raised. In addition, these variants also have several advantages, including the ability to increase the nonwovens’ mechanical capabilities and the aesthetic appearance of materials.

In contrast, comparable results are demonstrated on textured and twisted yarn fabrics with higher sound reduction than other staple yarn fabrics, especially II and III variants.

Taking under consideration plain fabric, the effectiveness of sound attenuation plummets as the number of layers increased both in variant II (TWP) and variant III fabrics TP, SP, TWP as well. Therefore, the sound absorption properties of the TP SP and TWP fabrics in II and III variants were higher than other investigated fabrics. The highest level of noise reduction (0.6) giving the lower reflection (40%) was achieved for a triple woven fabric TR, TS, TT, ST, TWR in the II variant. In addition, 0.61 of NRC results were demonstrated by triple woven rib fabric made of textured yarn together with nonwoven fabric.

The I variant generally showed that the tested materials could not be classified as sound absorber materials since their NRC values were less than 0.2, as specified in [29,30]. The second variant (II) results showed the greatest sound reduction compared to the other variants. Therefore, plain fabrics (TP, SP, TWP) could be classified as high-efficiency materials in variant II. Except for the SS weave structure, triple textiles with nonwoven demonstrated high-efficiency sound absorption. According to obtained results, woven fabrics made from textured yarn seemed to have a higher absorption rate than fabrics made from other yarn types in variant II, and therefore have a lower sound reflection. Finally, the III variant yielded somewhat lower results compared to the II variant. Nevertheless, all samples in III variant could be classified as useful acoustic material, because of the absorption coefficient values between 0.4 and 0.56, except for SR and SS.

Different weave structures exhibit varying degrees of sound attenuation capability. As previously stated, [20], the plain 1/1 fabric has a high degree of interlacement compared to the other weave types. Additionally, plain fabric thickness ranged between 0.4 and 0.6 mm, indicating that plain fabrics are thinner than other structures. TP (195 g/m^2^) and TWP (189 g/m^2^) have a lower mass per unit area than SP (213 g/m^2^). The low air permeability characteristics and acoustic test results from the anechoic chamber indicate that plain fabrics other than rib, sateen, and twill weave types have higher sound reduction properties. Additionally, the acoustic result from the impendence tube indicates that a single layer of plain weave structures has higher NRC results when combined with nonwoven and airgap. The rest of the weave structures exhibited a variable range of NRC values.

### 4.2. The Relationship between Physical Fabric Characteristics and Noise Reduction Coefficient of the Fabrics

The findings and discussion based on the surface roughness, porosity, and percentage of PES fiber in the fabric and their effect on sound absorption efficiency (Figure 3) are carried out. The NRC results are derived from Table 4. In Figure 3, the NRC is expressed in percentages for comparison with the physical characteristics of woven fabrics.

### 4.3. Roughness Result

The roughness test was conducted for both directions (weft and warp), from which the mean value was calculated. Plain weave structures such as TP, SP, and TWP have a low roughness (Figure 3, blue bar) compared to other weaves, followed by rib fabric structures TR, SR, and TWR. This phenomenon may have been connected to the degree of interlacement of the fabric structure. As the interlacement of warp and weft increased, it plunged the fabric roughness. This phenomenon is shown by the roughness values (Ra) (Figure 3, blue bar) of plain fabrics compared with other weave types. The comparison regarding yarn types shows that fabrics made with twisted yarn have a lower roughness than others.

The fabrics (single woven with air gap and nonwoven) TP, SP, and TWP showed high NRC and low surface roughness properties compared to other fabric types. On the other hand, the triple woven fabric (Table 4) TS showed the highest NRC properties together with high surface roughness (Figure 3). This phenomenon indicates that as the number of fabric layers increased, the fabric’s roughness effect became insignificant on the sound reduction properties.

It is possible to conclude, as the research results indicated, that the low fabric roughness showed high sound absorption of the single layer woven fabric. In addition, the increasing roughness of the fabric’s surface may increase the possibility of wave scattering from the surface. This phenomenon will cause the decreasing absorption of sound waves into the fabric. As a result, during the preparation of sound barrier materials, the effect of surface roughness on the sound absorption performance must be considered.

### 4.4. Percentage of PES Fiber Volume (Pv%)

Figure 3 (green bar) demonstrates that the amount of fiber in the fabric was dependent on the weave structure. In this case, the plain fabric’s structure contained a lot of fiber in comparison to others. The second-highest fiber content is found in the rib fabric structure. The percentage of fibers in sateen and twill fabrics are very similar. The relationship between noise reduction and fiber content of the fabric indicated that as the proportion of fiber in the fabric rises, the noise absorption improved. The TP, SP, and TWP fabrics verified this relationship as well.

### 4.5. Porosity Test Results

**Figure 4:**
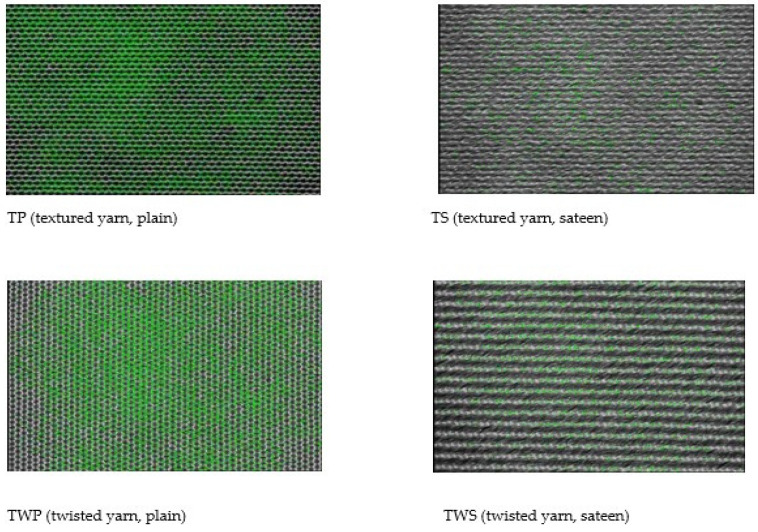
The distribution of porous surfaces on the fabric surface.

The outcome of the porosity test (Figure 3, purple bar) shows that the plain fabric structure has a large pore surface area compared to other weave types. Compared to other yarn types, the TP fabric has the most porous surface (11.4%). On the other hand, the sateen fabric generally presented a negligible porous surface, the sateen fabric (TWS) has the least porous space (0.4%) of all fabrics. The images presenting the highest and the lowest porous fabric surfaces are shown in Figure 4. The area of pores in fabric is colored green.

As compared to other weave structures, the plain fabric was highly interlaced. As the interlacement became greater, it increased the number of pores created between the warp and weft yarns. This porous space created a favorable surface for the entrance of sound waves to the structure. At that time, there was a possibility to change sound waves into energy during the friction between waves and particular fibers inside the yarn structure. Inversely, depending on the weave type, as the yarn floating in the fabric structure increased, the number of pores decreased simultaneously. This decreased the possibility of sound absorption by the material. As a result, the relationship between NRC and the fabric’s porosity indicated that TP, SP, TWP fabrics have highly porous surfaces (compared to the other fabrics) and high NRC results. In general, the high number of porosities in woven fabric creates a favorable environment for sound waves to penetrate and absorb inside the yarn structure.

### 4.6. The Acoustic Results, Based on the Range of Frequencies

Single-woven fabric sound absorption coefficient result comparison based on various measurement variants.

**Figure 5:**
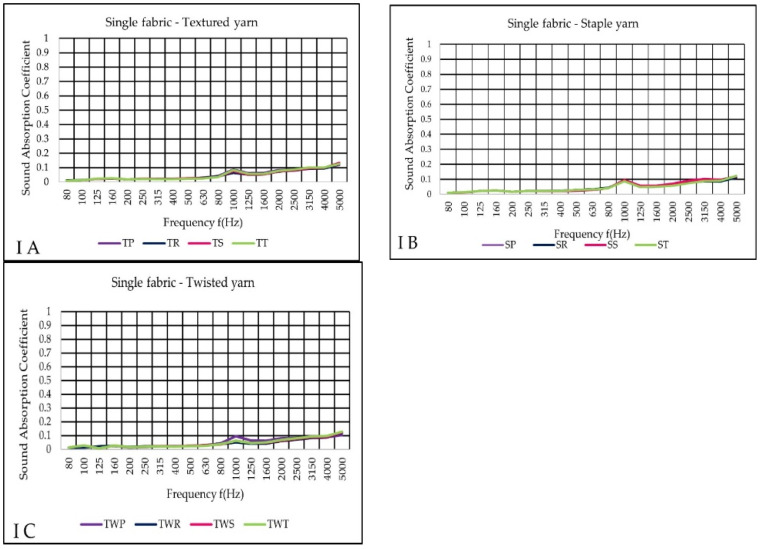
Sound absorption coefficient (α) versus frequency f (Hz) result of measurement variant (I) (**A**–**C**) single woven fabric.

The sound absorption coefficient (α) increased somewhat with frequencies in all fabrics (Figure 5A–C). The highest value α (0.1324) was verified in a sateen fabric (pink color) made of textured yarn (TS). The result of measurement A and B shows very similar absorption results at 1000 Hz. At the same frequency (1000 Hz) it showed that TWP fabric has a higher absorption point than others. However, tested single fabrics showed negligible sound absorption coefficient values, having nearly no effect on blocking, or absorbing sound waves.

The II variant of measurement findings included both single fabrics and nonwoven materials. Nonwoven fabric (orange color) has a maximum sound absorption of between 0.5 and 0.6 (α) at higher frequencies 2500–4000. The rest of the fabrics results demonstrated the combination of nonwoven and woven fabrics’ sound absorption capabilities. The outcome showed that very different results from variant (I).

**Figure 6:**
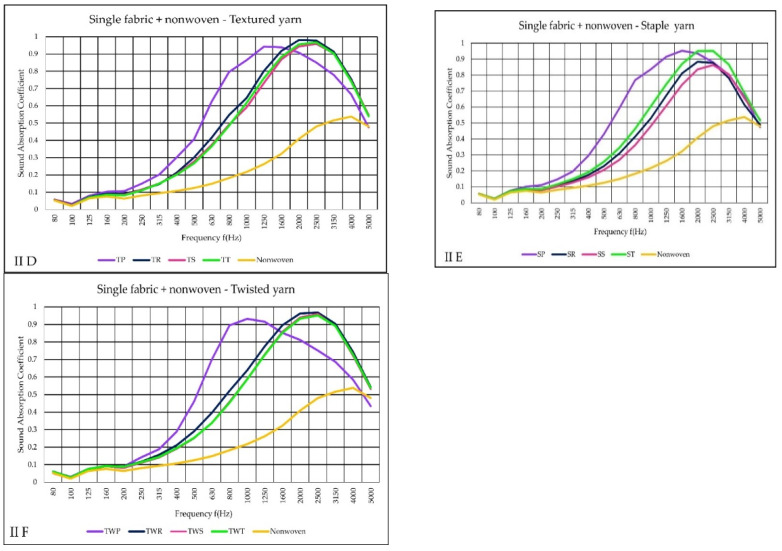
Sound absorption coefficient (α) versus frequencies f (Hz) results of II variant (**D**–**F**) single woven fabric + nonwoven.

Furthermore, plain fabrics (purple curve) presented different α results than other fabrics (Figure 6D–F). The increasing absorption point of plain fabrics (TP and SP) started at 200–250 Hz, reached the maximum at 1600 Hz.

This indicates that plain fabrics have a high sound absorption capacity at lower frequencies than other fabrics. For TWP however, the maximum absorption occurred at 1000 Hz. Higher sound absorption coefficients were demonstrated at high-frequencies ranges between 2000 and 2500 Hz.

The III variant (G–I) incorporated a 30 mm air gap between the solid plate and the fabric. The air gap sound absorption (blue curve) is below 0.1 (α). However, when the air gap followed woven fabric, their absorption performance went up significantly, in comparison to I variant. The air gap approach indicated that plain fabric could better absorb sound at low frequencies than other textiles. However, up 800 Hz, the outcome began to fluctuate.

At a frequency of 2000 Hz, the TP fabric has a higher absorption value, showing (α) 0.9. At 1250 Hz, the SP fabric demonstrated its superior sound absorption properties.

**Figure 7:**
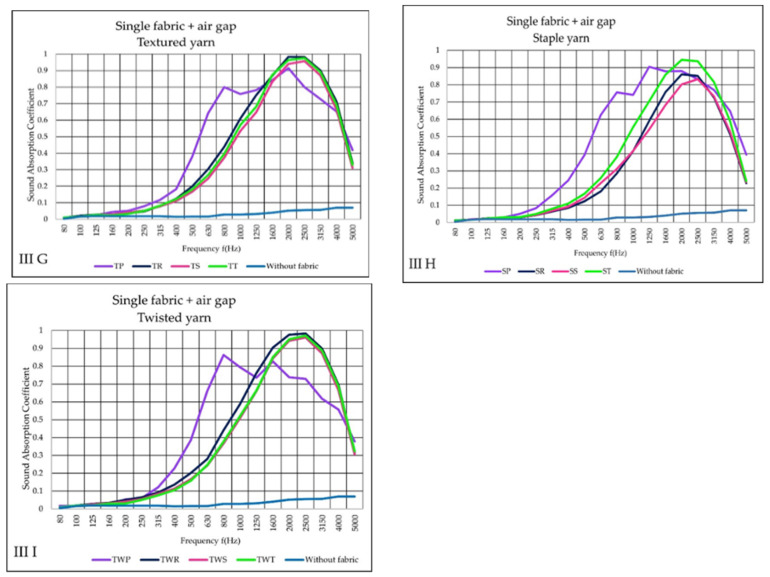
Sound absorption coefficient (α) versus frequency f (Hz) result of III variant, (**G**–**I**) single woven fabric + air gap.

Rib, sateen, and twill fabrics perform similar results, with the highest absorption level occurring at frequencies (2000–2500 Hz). Except for plain fabrics, textured and twisted yarn fabrics generally have a maximum sound absorption coefficient close to 1. Regarding staple yarn fabrics, ST has the second-highest absorption efficiency next to SP fabric. The third and least sound absorption were demonstrated on SR and SS fabric structures, as is shown in Figure 7 (III H).

Sound absorption coefficient outcome and discussion of double-woven fabrics measured in different variants.

**Figure 8:**
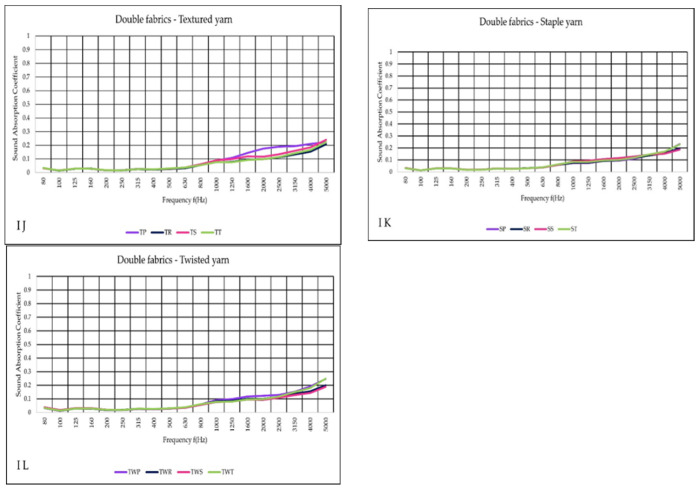
Sound absorption coefficient (α) versus frequency f (Hz) result of the variant (I), (**J**–**L**) double woven fabric.

The absorption of double fabric tested in I variant at lower frequencies was extremely low (Figure 8J–L). However, as the frequency range increased, the absorption of wave in double textiles showed improvement, beginning at 1000 Hz compared to the (I) single fabric measurement results. Between 1600 and 4000 Hz, TP (purple curve) increased absorption to the same extent as other textiles.

**Figure 9:**
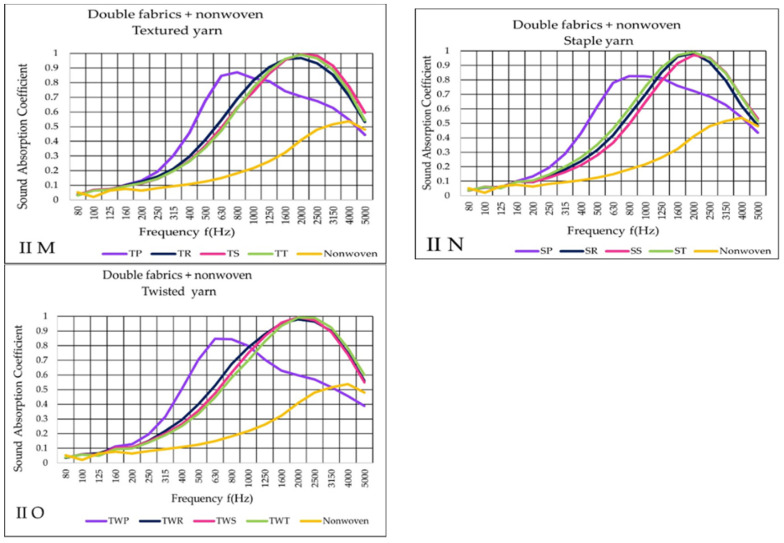
Sound absorption coefficient (α) versus frequency f (Hz) result of II variant (**M**–**O**) double woven fabric + nonwoven.

Figure 9M–O shows the substantial improvement of sound absorption for the II second variant (II). In contrast to other woven structures, plain fabrics exhibited increased sound absorption at low frequencies, similarly to previously presented I variant results. Additionally, the effect of reducing sound absorption was shown beginning at 1000 Hz. On the other hand, other tested woven fabrics in II variant textiles provided very comparable findings, with a maximum sound absorption coefficient approaching 1 at higher frequencies levels between 1600–3150 Hz. Finally, above >3150 Hz, the decreasing effect of the absorption is shown.

**Figure 10:**
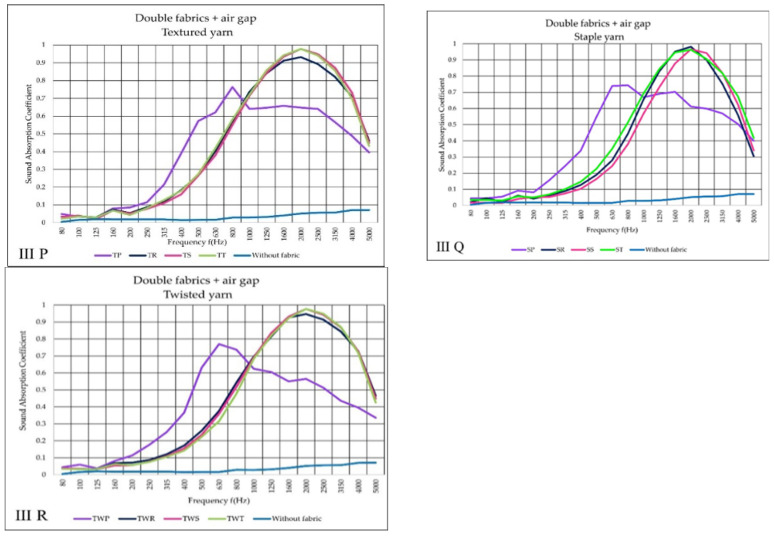
Sound absorption coefficient (α) versus frequency result f (Hz) of III variant (**P**–**R**) double woven fabric + air gap.

According to the findings of the III variant (Figure 10P–R) the plain fabric (purple curve) presented a lower sound absorption coefficient than tested in II variant. Air gap variant, on the other hand, enhanced the absorption of sound waves in low-frequencies. The maximum sound absorption of TP fabric occurred at 800 Hz. Other weaves provided quite similar results, reaching maximum sound absorption coefficient (α = 1) at 2000 Hz.

Sound absorption coefficient outcome and discussion of triple woven fabrics measured in different variants.

**Figure 11:**
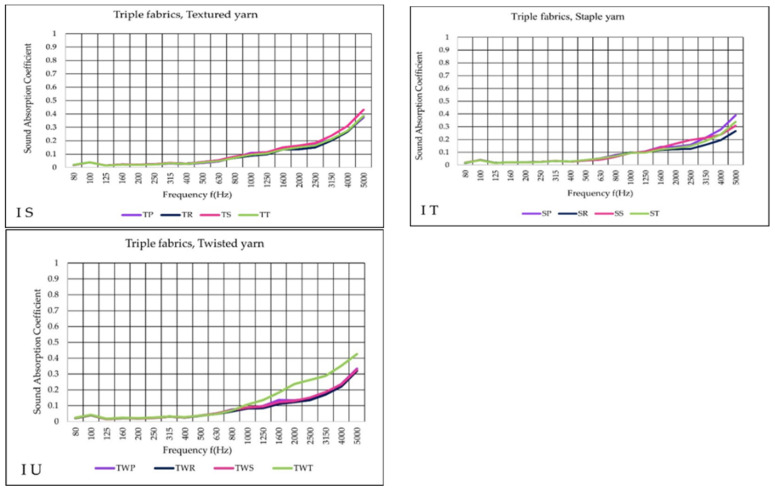
Sound absorption coefficient (α) versus frequency f (Hz) for result obtained in I variant (**S**–**U**) triple woven fabrics.

Figure 11S–U illustrates the performance of the three layers of woven sound absorption. As the layer count of the fabric increases, the absorption at higher frequencies increased too compared to the double fabrics tests. Different yarn types exhibited varying degrees of sound absorption. TS showed higher sound absorption results than the rest of the fabrics, comparison with the same yarn types. SP provided the best outcome when it comes to staple yarn fabrics. In terms of twisted yarn, TWT presented the leading result.

**Figure 12:**
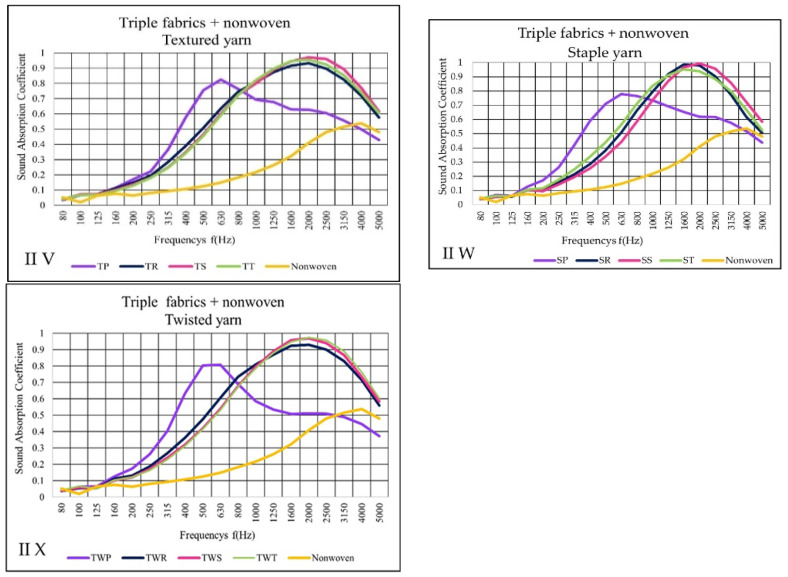
Sound absorption coefficient (α) versus frequency f (Hz) result of II variant (**V**–**X**) triple woven fabrics + nonwoven fabric.

The result of II variant (Figure 12V–X) shows that triple fabrics together with nonwoven gave the sound absorption coefficient, except for plain fabric, between 0.9 and 1 (α) from 1600 to 2500 Hz. These results were similar to double woven fabric in II variant results. Thus, plain fabrics exhibited a low sound absorption as woven fabric layers increase compared to the double layer in II variant. On the other hand, plain fabric structures absorbed low frequencies better than the other fabrics. 

**Figure 13:**
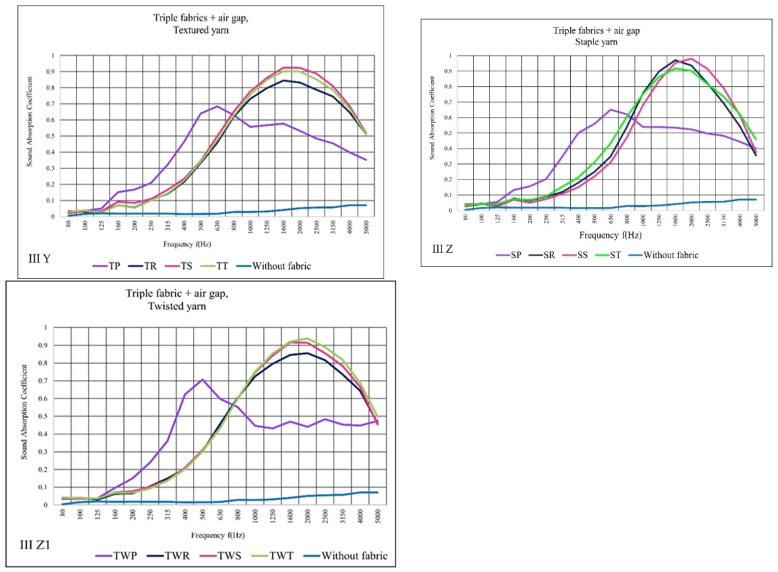
Sound absorption coefficient (α) versus frequency f (Hz) result of measurement III variant (**Y**–**Z1**) triple woven fabrics + air gap.

The triple fabric in the case of III variant results shows, that the plain fabrics were characterized by lower sound absorption than the double fabrics in III variant. TS, TT, TWS, and TWT fabrics show similar results for the maximum α (≥0.9) as in III variant too. SR and SS fabrics present an absorption coefficient close to 1 from 1600 to 2000 Hz (Figure 13).

In general, plain fabrics absorb more sound waves at lower frequencies than the rest of fabric structures. At higher frequencies, the absorption of other weave structures increases significantly. Depending on the variant’s comparison, the II variant (fabric with nonwoven) absorbs more sound than the I and III variants. On the other hand, the effect of an increasing layer of woven fabric has the following outcomes are demonstrated. Firstly, the sound absorption coefficients for just woven fabric are lower. As the number of layers of fabric increases, the sound absorption shows some improvements. Secondly, for plain fabrics, increasing the layer number, specifically with the nonwoven and air gap, decreases the sound absorption as increasing frequencies. Thirdly, except for plain fabric with nonwoven and air gap, increasing the number of woven fabric layers has no significant effect, and nearly identical results were achieved between 0.9 and 1α.

## 5. Summary and Conclusions

This study compares the sound absorption efficiency of various layers of woven textiles measured according to three variants, such as woven fabric (variant I), woven fabric with nonwoven (variant II), and woven fabric with an air gap (variant III). Furthermore, the impact of fabric physical properties on sound absorption of woven textiles, such as surface roughness, porosity, and PES fiber content in the fabric, was investigated. The results are as follows:

- The noise reduction coefficient (NRC) results enable us to compare the absorption capacity of various fabrics. Textiles made of textured and twisted yarn show comparable NRC findings for variants II and III and greater absorption than staple yarn fabrics except for the plain fabric and ST. In variants II and III, plain fabrics have a greater sound absorption capacity than other fabric structures, particularly single layer woven fabric and double woven TP fabric. Variant I result cannot be considered an absorber material due to the NRC being below 0.2. The second (II) variant achieves a significant reduction in noise than the I and II variant. The III variant shows intermediate results that are much higher than those of the I variant and lower than those of the II variant.
- When the surface roughness of the woven fabric is reduced, the noise reduction coefficient performance of the fabric rises. This phenomenon is directly related to the possibility of wave scattering from the surface as an increase in roughness. Furthermore, higher PES fiber content and porosity indicate that plain fabrics have higher noise reduction coefficient results than other weaves such as rib, sateen, and twill.
- In general, based on the sound absorption coefficient, the I variant, sound absorption (α), demonstrates no sound absorption properties. On the other hand, apart from plain fabrics, the sound absorption coefficient of all fabrics in II variant is between 0.9 and 1 (α) at higher frequencies (1600 to 2500 Hz). Similarly, plain fabrics’ sound absorption coefficient (α) decreases as the number of woven fabric layers rises in variant II (TWP), and in variant III, TP, SP, and TWP. However, these fabrics have a more excellent absorption at low frequencies (200 to 1000 Hz) than the others.
- Combining woven and nonwoven fabrics has advantages in improving sound barrier properties. The first is to make the sound absorption performance better for both textiles. Secondly, integrated materials may have better mechanical characteristics than nonwoven itself. Thirdly, the aesthetic value of such mixed materials can be achieved by giving elaborated woven fabrics as a front layer. Generally, the absorption improvement at low frequencies for single layer plain fabrics together with nonwoven or air gaps gives a clue for further investigation into developing such materials for the application of low sound absorber materials.

## Figures and Tables

**Table 1:** Fabric construction parameters [20].

Yarn Type	Weave Type	Sample Code	Warp Density (Threads/cm)	Weft Density (Threads/cm)	Mass Per Unit Area, (g/m^2^)
Textured yarn	Plain	TP	31 ± 0	18 ± 0	195 ± 1.5
	Rib	TR	38 ± 0	17 ± 0	224 ± 2.8
	Sateen	TS	34 ± 0.4	20 ± 0.6	213 ± 2.1
	Twill	TT	32 ± 0	18 ± 0.6	210 ± 4.2
Staple yarn	Plain	SP	32 ± 0.2	17 ± 0.6	213 ± 1.6
	Rib	SR	35 ± 0	17 ± 0.5	211 ± 1.5
	Sateen	SS	33 ± 1.1	16 ± 0	202 ± 0.5
	Twill	ST	33 ± 0.2	18 ± 0	210 ± 3.3
Twisted yarn	Plain	TWP	31 ± 0	17 ± 0.8	189 ± 0.75
	Rib	TWR	35 ± 0.1	16 ± 0.6	203 ± 2
	Sateen	TWS	35 ± 0	17 ± 1	195 ± 0.63
	Twill	TWT	34 ± 0.2	20 ± 1	197 ± 0.8

**Table 2:** Measurement variants inside the impedance tube.

Position of Tested Sample Inside the Impedance Tube	
Variant I. Woven fabric	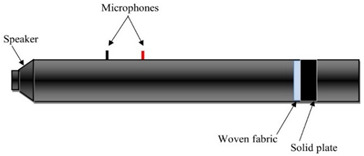
Variant II. Woven fabric + nonwoven	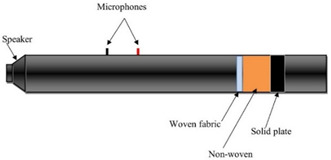
Variant III. Woven fabric + air gap	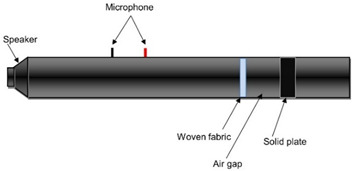

**Table 3:** Arrangement and setting of woven fabrics.

Arrangement and Set of Woven Fabrics		
Woven fabric	Double woven fabrics	Triple woven fabrics
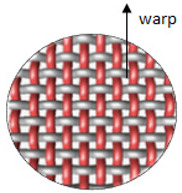	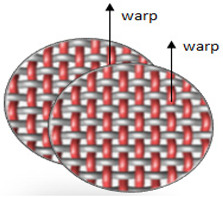	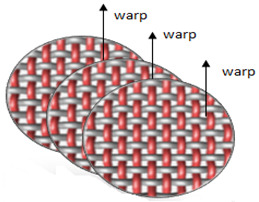

**Table 4:** Percentage of noise reduction coefficient (NRC) of the samples.

	Variant I			Variant II			Variant III		
Sample Code	Single Woven Fabric NRC/Reflection %	Double Woven Fabric NRC/Reflection %	Triple Woven Fabric NRC/Reflection %	Single Woven + Nonwoven NRC/Reflection %	Double Woven Fabric + Nonwoven NRC/Reflection %	Triple Woven Fabric + Nonwoven NRC/Reflection %	Single Woven Fabric + Air Gap NRC/Reflection %	Double Woven Fabric + Air Gap NRC/Reflection %	Triple Woven Fabric + Air Gap NRC/Reflection %
TP	0.05/95	0.08/92	0.08/92	0.58/42	0.6/40	0.57/43	0.53/47	0.49/51	0.48/52
TR	0.05/95	0.05/95	0.07/93	0.51/49	0.59/41	0.61/39	0.46/54	0.51/49	0.5/50
TS	0.05/95	0.06/94	0.08/92	0.48/52	0.56/44	0.6/40	0.42/58	0.51/49	0.54/46
TT	0.05/95	0.06/94	0.08/92	0.49/51	0.56/44	0.6/40	0.44/56	0.51/49	0.52/48
SP	0.05/95	0.05/95	0.07/93	0.59/41	0.59/41	0.58/42	0.52/48	0.5/50	0.46/54
SR	0.05/95	0.06/94	0.07/93	0.44/56	0.53/47	0.58/42	0.36/64	0.47/53	0.51/49
SS	0.05/95	0.06/94	0.08/92	0.41/59	0.51/49	0.55/45	0.35/65	0.44/56	0.49/51
ST	0.05/95	0.06/94	0.07/93	0.48/52	0.56/44	0.6/40	0.43/57	0.49/51	0.51/49
TWP	0.05/95	0.06/94	0.07/93	0.59/41	0.57/43	0.56/44	0.49/51	0.5/50	0.46/54
TWR	0.04/96	0.06/94	0.07/93	0.5/50	0.58/42	0.6/40	0.46/54	0.5/50	0.5/50
TWS	0.04/96	0.05/95	0.07/93	0.47/53	0.56/44	0.59/41	0.42/58	0.5/50	0.52/48
TWT	0.04/96	0.06/94	0.1/90	0.47/53	0.54/46	0.58/42	0.42/58	0.49/51	0.52/48

## Data Availability

The data presented in this study are available by request to the corresponding author.
